# Non-geodesic timelike observers and the ultralocal limit

**DOI:** 10.1140/epjc/s10052-025-13888-6

**Published:** 2025-02-12

**Authors:** Reza Saadati, Luca Valsan, Valerio Faraoni

**Affiliations:** 1 Department of Physics and Astronomy, Bishop’s University, 2600 College Street, Sherbrooke, QC J1M 1Z7 Canada; 2 Department of Physics, McGill University, 3600 rue University, Montreal, QC H3A 2T8 Canada

## Abstract

The ultralocal limit along timelike geodesics, in which any geometry reduces to Bianchi I, does not extend to non-geodesic timelike observers. Exceptions are discussed, including particles with variable mass, test particles in Einstein frame scalar-tensor gravity, and self-interacting dark matter.

## Introduction

Ultralocal limits in general relativity (GR) have been the subject of renewed interest in recent years. In an ultralocal limit, one restricts oneself to looking at the 3-space in an infinitesimally small neighborhood of a null or timelike geodesic. In physical terms, in an ultralocal limit in which the light cones close and collapse on the observer’s wordline, nothing propagates in its 3-space. This situation is often pictured as saying that in this limit the speed of light *c* tends to zero. This is the Carrollian limit of GR [1–3], the opposite situation to Newtonian physics in which the light cones instead open up and flatten out on the 3-space of the observer, while *c* tends to infinity. The ultra-relativistic Carroll limit, originally studied as a formal limit of the Poincaré algebra, is associated with quantum effects in strong gravity regimes and the AdS/CFT correspondence (e.g., [4–6]). While, in reality, *c* is a constant of nature with a definite value, considering these limits makes sense physically.

Almost half a century ago, Penrose demonstrated that along null geodesics every spacetime metric reduces to a plane wave in the ultralocal limit [7]. This work was related to the importance of *pp*-waves [8] and can be interpreted physically by saying that, for a freely falling observer reaching asymptotically the speed of light, any gravitational field would look that of an exact plane gravitational wave passing by at light speed. An important feature of this result is its universality.

Another known application of the ultralocal limit is the study of the approach to spacelike singularities: Belinski, Khalatnikov and Lifshitz [9, 10] showed that in the ultra-local limit, the vacuum geometry near a spacelike singularity is approximated by a Kasner one with line element

1 $$\begin{aligned} ds^2 =- dt^2 + \left( a_1 t^{p_1} \right) ^2 dx^2 + \left( a_2 t^{p_2} \right) ^2 dy^2 + \left( a_3 t^{p_3} \right) ^2 dz^2 \end{aligned}$$

with $$ p_1 + p_2 + p_3=1 $$, $$ p_1^2 + p_2^2 + p_3^2 =1$$ and constant $$a_i$$  ($$i=1,2,3$$). This is a special case of a Bianchi I geometry, with the exponents $$p_i$$ depending on the spatial coordinates, but this dependence vanishes in the ultralocal limit.

More recently, Cropp and Visser [11, 12] worked out a similar limit for geodesic *timelike* observers, which is a more physical situation. They demonstrated that freely falling observers see *any* gravitational field as a Bianchi I model in the ultralocal limit [11, 12]. Here the Cropp–Visser ultralocal limit is revisited and it is shown that timelike *non-geodesic* observers do not enjoy such a universal ultralocal limit, but that there are certain physically relevant exceptions to this rule.

In general, the Cropp–Visser ultralocal limit fails for non-geodesic timelike curves because the very first step, the introduction of Gaussian normal (or “synchronous”) coordinates, is not possible along non-geodesic curves. However, there are very special and restricted situations in which an accelerated particle is subject to a four-force but the discussion of [11, 12] still applies in the ultralocal limit and the geometry looks again like that of a Bianchi I universe to this particle. This situation occurs when the four-force acting on the observer is tangential to its trajectory (i.e., parallel or anti-parallel to its four-tangent $u^\mu$), in which case synchronous coordinates can still be introduced and the derivation of the ultralocal limit in [11, 12] proceeds as for geodesic timelike curves.

In the following sections we recall the standard derivation of the ultralocal limit of [11, 12] (Sect. 2), showing explicitly why the procedure fails for non-geodesic curves (Sect. 3); we then extend the validity of the Cropp–Visser limit to non-geodesic timelike curves with four-force parallel to the curve (Sect. 4). Section 5 muses about generic accelerated observers, while Sect. 6 summarizes the results.

We follow the notation of Ref. [13]. Units are used in which the speed of light *c* and Newton’s constant *G* are unity. Greek (spacetime) indices run from 0 to 3, while (purely spatial) Latin indices run from 1 to 3, $g_{\mu \nu }$ denotes the spacetime metric and $\nabla _{\alpha }$ the corresponding covariant derivative.

## The ultralocal limit for timelike geodesics

We report briefly the Cropp–Visser derivation [11]. One begins by assuming an observer freely falling along a timelike geodesic $\gamma$ of the spacetime metric $g_{\mu \nu }( x^{\alpha })$. The first step consists of adopting synchronous (or Gaussian normal) coordinates, which are always defined locally in the neighborhood of a timelike geodesic curve. Locate the origin of the spatial coordinates $x^i$ at the curve $\gamma$. In these coordinates, the line element assumes the form

2 $$\begin{aligned} ds^2=-c^2dt^2 + g_{ij}\left( t, x^k \right) dx^i dx^j , \end{aligned}$$

i.e., $g_{00}=-1$ and $g_{0i}=0$ in these coordinates. Now consider the coordinate transformation

3 $$\begin{aligned} x^i\rightarrow x^{i'}( x^j) = \varepsilon \, x^i , \end{aligned}$$

where $\varepsilon$ is a positive constant, which involves only a rescaling of the spatial coordinates. We will be interested in the limit $\varepsilon \rightarrow 0$, which clearly corresponds from the outset to shrinking the 3-space around the worldline $\gamma$ onto the worldline itself. The line element has taken the form

4 $$\begin{aligned} ds^2= -c^2dt^2 + \varepsilon ^2 g_{ij}\left( t, \varepsilon \, x^k \right) dx^i dx^j \,; \end{aligned}$$

now rescale the speed of light as $c\rightarrow \varepsilon \, c$, obtaining

5 $$\begin{aligned} ds^2=\varepsilon ^2 \left[ -c^2dt^2 + g_{ij}\left( t, \varepsilon \, x^k \right) dx^i dx^j \right] , \end{aligned}$$

and perform the conformal transformation $ds^2 \rightarrow d\tilde{s}^2 = \varepsilon ^{-2} ds^2$ (as is done also for the Penrose limit along null geodesics [7]), which gives

6 $$\begin{aligned} ds^2= -c^2dt^2 + g_{ij}\left( t, \varepsilon \, x^k \right) dx^i dx^j . \end{aligned}$$

Finally, we take the limit $\varepsilon \rightarrow 0$, obtaining the line element

7 $$\begin{aligned} ds^2= -c^2dt^2 + g_{ij}\left( t, \textbf{0} \right) dx^i dx^j , \end{aligned}$$

which has the form describing a Bianchi I universe [14, 15].

## The failure of synchronous coordinates and of the universal ultralocal limit

Let us ask now the question of how the spacetime geometry will appear to an *accelerated* timelike observer. Unfortunately, the derivation of the result that it appears as a Bianchi I universe fails at its very first step: synchronous coordinates cannot be introduced along non-geodesic timelike curves. To see why, let us examine how the standard derivation of Gaussian normal coordinates (e.g., [13]) is modified by the fact that these curves deviate from geodesics, and what the obstruction is precisely.

Assume that a massive particle is subject to a four-force per unit mass $f^{\mu }$ and follows the spacetime trajectory $\gamma '$ with timelike tangent $u^{\mu }$ described by the equation

8 $$\begin{aligned} u^{\nu } \nabla _{\nu } u^{\mu }= \frac{du^{\mu }}{d\tau } + \Gamma ^{\mu }_{\alpha \beta } \, u^{\alpha } u^{\beta } =f^{\mu } , \end{aligned}$$

where $\tau$ is the proper time along the trajectory and $\Gamma ^{\mu }_{\alpha \beta }$ are the Christoffel symbols of the connection. To introduce synchronous coordinates $\left( \tau , x^i \right)$, the wordline $\gamma '$ of the particle must be normal to every hypersurface of 3-space $\Sigma _{\tau }$ of constant time $\tau$, in which there are spatial coordinates $x^i$ with three purely spatial coordinate vectors $X^{\nu }$ according to the observer $u^\mu$. That is, for all $\Sigma _{\tau }$, we must have $u^{\mu } X_{\mu } =0$ for each of the three vectors $X^{\alpha }$. This means that, defining the spatial coordinates so that $X^{\mu } u_{\mu }=0$ initially, this condition is preserved along $\gamma '$, i.e.,

9 $$\begin{aligned} \frac{D}{D\tau } \left( X^{\mu } u_{\mu } \right) = u^{\nu } \nabla _{\nu } \left( X_{\mu } u^{\mu } \right) =0 , \end{aligned}$$

but this property fails to hold because it is instead

10 $$\begin{aligned} u^{\nu } \nabla _{\nu } \left( X_{\mu } u^{\mu } \right) = X_{\alpha } f^{\alpha }. \end{aligned}$$

To wit,

11 $$\begin{aligned} u^{\nu } \nabla _{\nu } \left( X_{\alpha } u^{\alpha } \right)= &   u_{\alpha } u^{\beta } \nabla _{\beta } X^{\alpha } +X^{\alpha } u^{\beta } \nabla _{\beta } u^{\alpha } \nonumber \\= &   u_{\alpha } u^{\beta } \nabla _{\beta } X^{\alpha } +X_{\alpha } f^{\alpha } . \end{aligned}$$

Since $u^{\mu }$ and $X^{\mu }$are the vectors of a coordinate basis on the spacetime manifold their commutator vanishes, yielding

12 $$\begin{aligned} u^{\beta } \nabla _{\beta } \left( X_{\alpha } u^{\alpha } \right) = u_{\alpha } X^{\beta } \nabla _{\beta } u^{\alpha } +X_{\alpha } f^{\alpha } = X_{\alpha } f^{\alpha } \end{aligned}$$

because the normalization $u^{\mu } u_{\mu }=-1$ implies that $u^{\alpha } \nabla _{\beta } u_{\alpha } =0$.

The right-hand side of Eq. (12) vanishes if and only if the particle is free or is subject to a four-force perpendicular to $X^{\mu }$. A clear obstruction is thus identified in constructing synchronous coordinates along non-geodesic timelike curves.

## Exceptions

If the four-force $f^{\mu }$ is parallel or antiparallel to the four-velocity $u^{\alpha }$, the product $X_{\alpha } f^{\alpha }$ vanishes, Eq. (12) reduces to the affinely parametrized geodesic equation, and the obstruction to constructing synchronous coordinates is removed.

The condition that the four-force acting on a massive particle be (anti-)parallel to the particle trajectory’s tangent seems rather exceptional, to the point that is not contemplated in most textbooks. However, this circumstance is far from unphysical, including situations in which the particle mass changes along the trajectory, the ultralocal limit of particles subject only to gravity in the Einstein frame description of scalar-tensor gravity [16], fluid elements of perfect or imperfect fluids in Friedmann–Lemaître–Robertson–Walker (FLRW) or Bianchi universes, mass-changing particles in cosmology and in scalar-tensor gravity [17–22], and certain scenarios in which self-interacting dark matter is effectively subject to a sort of anti-friction [23]. In the standard textbook treatment, the mass *m* of a particle following a timelike worldline $\gamma '$ with four-tangent $u^\mu$ is constant and the four-force acting on it is simply $f^{\alpha }=m a^{\alpha }$, where $a^{\beta } \equiv \dot{u}^{\beta } \equiv u^{\mu } \nabla _{\mu } u^{\beta }$ is the particle’s four-acceleration. Rockets are prototypical systems with variable mass and exact solutions describing rockets [15, 24–33] and solar sails [34–36] abound in GR.

Another instance of four-force parallel to a particle trajectory arises in cosmology. Quantum processes in the early universe lead to particle production and cause a negative bulk pressure [37, 38]. This mechanism could potentially drive inflation, as suggested in [23, 39–43]. Likewise, the self- interaction of dark matter can cause negative bulk stresses, a mechanism currently under investigation as a possible cause of the present acceleration of the universe [23]. Indeed, this self-interaction would be responsible for a cosmic “antifriction” on the dark matter fluid, i.e., for a force antiparallel to the timelike four-trajectories of dark matter particles [23].

Yet again, in (Jordan frame) scalar-tensor gravity [44–50], a scalar field degree of freedom $\phi$ (the Brans–Dicke-like scalar) appears together with the two massless spin two modes contained in the metric tensor in GR. The action is

13 $$\begin{aligned} S_\textrm{ST}= &   \frac{1}{16\pi } \int d^4 x \, \sqrt{-g} \, \left[ \phi R -\frac{\omega (\phi )}{\phi } \, \nabla ^{\mu } \phi \nabla _{\mu } \phi -V(\phi ) \right. \nonumber \\  &   \left. +\mathcal{L}^\mathrm {(m)} \right] , \end{aligned}$$

where *g* is the metric determinant, *R* is the Ricci scalar, $\omega (\phi )$ is the Brans–Dicke coupling, $V(\phi )$ is a potential for the scalar field, and $\mathcal{L}^\mathrm {(m)}$ is the matter Lagrangian density. The Jordan frame variables are $\left( g_{\mu \nu }, \phi \right)$, Newton’s constant *G* is replaced by the effective gravitational coupling strength $G_\textrm{eff}\simeq 1/\phi$, and $\phi$ couples directly to the Ricci scalar *R*. In the Einstein frame representation of these theories, the variables $\left( \tilde{g}_{\mu \nu }, \tilde{\phi } \right)$ are defined by

14 $$\begin{aligned}  &   \tilde{g}_{\mu \nu } = \phi \, g_{\mu \nu } , \end{aligned}$$

15 $$\begin{aligned}  &   d\tilde{\phi } =\sqrt{ \frac{2\omega +3}{16\pi }} \, \frac{d\phi }{\phi } . \end{aligned}$$

In this frame the scalar $\tilde{\phi }$ does not couple explicitly to the spacetime curvature, but couples directly to matter and the action (13) is rewritten as

16 $$\begin{aligned} S_\textrm{ST}= &   \int d^4 x \, \sqrt{-\tilde{g}} \, \left[ \frac{ \tilde{R}}{16\pi } -\frac{1}{2} \, \tilde{g}^{\mu \nu } \tilde{\nabla }_{\mu } \tilde{\phi } \tilde{\nabla }_{\nu } \tilde{\phi } -U( \tilde{\phi }) \right. \nonumber \\  &   \left. + \frac{\mathcal{L}^\mathrm {(m)}}{ \phi ^2(\tilde{\phi })} \right] \end{aligned}$$

in terms of the Einstein frame variables $\left( \tilde{g}_{\mu \nu } \tilde{\phi } \right)$, where

17 $$\begin{aligned} U \left( \tilde{\phi }\right) = \frac{ V(\phi )}{ \phi ^2} \Big |_{\phi =\phi ( \tilde{\phi }) }. \end{aligned}$$

As a consequence particles subject only to gravity, which follow geodesics in the Jordan frame, deviate from geodesics in the Einstein frame, according to [51, 52]

18 $$\begin{aligned} \frac{d^2 x^{\mu }}{d\tau ^2} + \tilde{\Gamma }^{\mu }_{\alpha \beta } \, \frac{dx^{\alpha }}{d\tau } \, \frac{dx^{\beta }}{d\tau } = \sqrt{\frac{4\pi }{2\omega +3}} \, \tilde{\nabla }^{\mu } \tilde{\phi }. \end{aligned}$$

The common interpretation of this equation is that the mass of a test particle (which was constant in the Jordan frame) instead depends on $\tilde{\phi }$ in the Einstein frame and is no longer geodesic.^1^ The gradient of $\phi$ across spacetime translates in the dependence of the particle mass *m* on the spacetime position and in a fifth force proportional to $\tilde{\nabla }^{\mu } \tilde{\phi }$ [16, 52].^2^

Once the particle trajectory $\gamma '$ is fixed by initial conditions, the particle mass depends only on the proper time $\tau$ along this trajectory. In the ultralocal limit, the spatial dependence of $\phi$ is killed anyway, leaving $\phi \left( t, \textbf{0} \right)$ instead of $\phi \left( t, \textbf{x} \right)$, and $m=m(\tau )$. In these conditions, one effectively has a time-dependent mass along the trajectory and a four-force $f^\mu$ parallel to its four-tangent $u^\mu$. The conclusion that test particles subject only to gravity “see” ultra-locally any spacetime geometry as a Bianchi I geometry, therefore, holds for Einstein frame scalar-tensor gravity.

Another GR situation in which a four-force parallel to massive particle worldlines occurs in cosmology. Consider a FLRW universe sourced by a fluid, with line element

19 $$\begin{aligned} ds^2 =-dt^2 +a^2(t) \left[ \frac{dr^2}{1-kr^2} +r^2 \left( d\vartheta ^2 +\sin ^2\vartheta \, d\varphi ^2 \right) \right] \end{aligned}$$

in comoving coordinates $\left( t, r, \vartheta , \varphi \right)$. Except for the case in which the matter fluid is dust or a cosmological constant $\Lambda$ (corresponding to stress-energy tensor $T_{\mu \nu }^{(\Lambda )}=-\frac{\Lambda }{8\pi } \, g_{\mu \nu }$), this fluid has pressure *P*(*t*) and pressure gradient $\nabla _{\mu } P \ne 0$, which generates a four-force pointing in the (comoving) time direction $u^\mu$. In fact, the four-force and four-acceleration must have vanishing spatial components to respect spatial isotropy. As a consequence, fluid particles are accelerated and deviate from geodesics. The fluid particles obey the equation (e.g., [16])

20 $$\begin{aligned} \frac{d^2 x^{\mu }}{dt^2} + \Gamma ^{\mu }_{\alpha \beta } \, \frac{dx^{\alpha }}{dt} \, \frac{ dx^{\beta }}{dt} = B \, \frac{dx^{\mu }}{dt} , \end{aligned}$$

where *B* depends on the position along the particle worldline. This equation is just the non-affinely parametrized geodesic equation, where the proper time *t* of comoving observers is not an affine parameter. It is always possible to switch from *t* to an affine parameter: then the right-hand side of the geodesic equation (20) vanishes. We conclude that this four-acceleration is somehow trivial, but the reparametrization is not. If *s* is an affine parameter, the function *B* in Eq. (20) is $B(t)= \frac{dt}{ds} \, \frac{d^2\,s}{dt^2}$ ([14, 16], see Appendix A of [56]). The equation describing the worldlines of the fluid elements cannot be affinely parameterized by the fluid’s proper time, which causes a four-force parallel to the four-velocity $u^\mu$ [16, 56]. From the purely mathematical point of view, this fact is immaterial but the difference between the proper time of the comoving observers and an affine parameter matters from the physical point of view. FLRW cosmology is always described using the frame of comoving observers.

This discussion extends to Bianchi cosmologies, reaching the conclusion that pressure gradients and anisotropic stresses generate four-forces parallel to the fluid worldlines, therefore the ultralocal limit applies [56]. This fact can perhaps be taken as a consistency check of the ultralocal limit for non-geodesic fluids, but is almost trivial since it states that a FLRW or Bianchi universe (possibly with spatial curvature) looks locally like a Bianchi I spacetime! FLRW is a special case of Bianchi I with vanishing spatial anisotropy and any spatially curved Bianchi model reduces to Bianchi I when the spatial curvature is negligible in the local limit.

## General accelerated observer

Since, in the general case, one cannot introduce synchronous coordinates along the wordline $\gamma '$ of an accelerated observer, the best on can do to perform an ultralocal limit is the following. Given the force $f^\mu$ acting on an accelerated timelike observer and it worldline $\gamma '$ satisfying Eq. (8), one can perform a rescaling of the spatial coordinates in the 3-space with Riemannian metric $h_{\mu \nu }= g_{\mu \nu } + u_{\mu } u_{\nu }$ and then take the limit $\varepsilon \rightarrow 0$ as described in Sect. 2, obtaining

21 $$\begin{aligned} ds^2= &   g_{00} \left( t(\tau ), \textbf{0} \right) dt^2 + 2 g_{0i} \left( t(\tau ), \textbf{0} \right) dt dx^i \nonumber \\&\,&+ g_{ij} \left( t(\tau ), \textbf{0} \right) dx^i dx^j , \end{aligned}$$

where $t(\tau )=x^0(\tau )$ is the time component of the solution $x^{\mu }(\tau )$ of Eq. (8). One can then redefine the time coordinate $t \rightarrow \bar{t}$ according to $-g_{00} \left( t(\tau ), \textbf{0} \right) dt^2 =d\bar{t}^2$, or

22 $$\begin{aligned} \bar{t}= \int d\tau \, \frac{dt}{d\tau }\, \sqrt{-g_{00}\left( t(\tau ) , \textbf{0} \right) }. \end{aligned}$$

$d\bar{t}$ is an exact differential because the integrand in Eq. (22) depends only on $\tau$. We are left with the time-dependent geometry

23 $$\begin{aligned} ds^2 = - d\bar{t}^2 + 2 \bar{g}_{0i} \left( \bar{t} \right) d\bar{t} dx^i + g_{ij} \left( \bar{t} \right) dx^i dx^j , \end{aligned}$$

where $\bar{g}_{0i} \left( \bar{t} \right) = g_{0i} \left( t(\tau (\bar{t}) ), \textbf{0} \right)$, $\bar{g}_{ii} \left( \bar{t} \right) = g_{ij} \left( t(\tau (\bar{t}) ), \textbf{0} \right)$ are obtained by inverting the relation $\bar{t}(\tau )$. This geometry is still very general and the universality of the ultralocal limit for geodesic observers (in which *any* geometry looks like Bianchi I) is completely lost.

One could pose the problem of whether a prescribed geometry (23) can be obtained by tailoring the four-force $f^\mu \left( x^{\alpha }, \dot{x}^{\alpha } \right)$ acting on an accelerated observer. However, this problem is not amenable to mathematical treatment. Even if Eq. (8) could be solved analytically and explicitly for all forces $f^\mu$ deemed necessary to achieve a required geometry $\bar{g}_{\mu \nu }(\bar{t})$ (which is in practice impossible), the solution would comprise only four functions $x^{\mu } (\tau )$ while specifying completely the metric components $\bar{g}_{0i}, \bar{g}_{ij}$ requires one to impose nine conditions. There is no precise mathematical way to relate these nine functional relations (or a subset of four of them) to the force $f^\mu$, and no way to pose a mathematically well-defined problem. It may well be that in some special cases a solution to this problem exists: for example, thanks to Ref. [11] we know that the problem of imposing $\bar{g}_{\mu \nu }$ to be of the Bianchi I form has a solution for $f^{\mu }=0$ (we do not know whether this is the unique solution). However, in general, no solution to this problem posed vaguely in mathematical terms can be expected.

## Conclusions

The ultralocal limit along timelike geodesics, in which all geometries look Bianchi I to freely falling observers, does not extend to the non-geodesic timelike curves followed by accelerated particles, with the exception of particles subject to a four-force parallel (or antiparallel) to the four-tangent to the trajectory. Although quite special, this situation should not be dismissed *a priori* as unphysical or irrelevant. As already noted, the case of particles subject to pressure gradients in cosmology is trivial because one starts with cosmology and recovers a cosmology in the ultralocal limit, but other situations are by all means not trivial. They include particles with variable mass (e.g., rockets and solar sails, which are the subject of a non-negligible literature in GR [15, 24–36]), test particles in Einstein frame scalar-tensor gravity, and self-interacting dark matter particles in certain scenarios [23]. The fact that the ultralocal limit extends to scalar-tensor gravity comes to no surprise since the field equations are not used in the derivation of the ultralocal limits [7, 11, 12].

We have in mind an application of the ultralocal limit to a new thermal view of scalar-tensor (including “viable” Horndeski) gravity [56–76] in the case in which the gradient $\nabla ^{\mu } \phi$ of the gravitational scalar field is timelike and future-oriented. In this formalism, the effective stress-energy tensor of $\phi$ has the form of a dissipative fluid stress-energy tensor and satisfies Eckart’s [77] generalization of the Fourier law of heat conduction, which leads one to defining a “temperature of gravity”. Then, the approach of scalar-tensor gravity to general relativity is analogous to the dissipation of heat in a medium; the relaxation of gravity to general relativity is described as the approach of a heated medium to its zero-temperature equilibrium state. This new formalism is still under development, but Bianchi I models have been discussed in this context [74]. The fact that in the ultralocal limit all scalar-tensor spacetimes are seen as Bianchi I, and the reduction of Einstein frame non-geodesic observers to geodesic ones discussed in this work, has several potential implications for the new thermal view of scalar-tensor gravity. Among them, we mention the following: it is possible that non-viable Horndeski gravity (i.e., the class of theories in which the speed of gravitational waves is not equal to the speed of light), which could not be included in the thermal view, becomes tractable in the ultralocal limit because of the simplifications that this limit brings to the effective fluid quantities. This application will be discussed in future work.

## Data Availability

This manuscript has no associated data. [Author’s comment: All relevant data are within the paper and its Supporting Information files.]

## Footnotes

1 However, since the scalar field $\phi$ has gravitational nature, this particle is still subject only to gravity and to no other forces, but now gravity is described by *both* $g_{\mu \nu }$ and $\phi$.

2 In dilaton gravity and in the low-energy limit of string theories, a similar equation appears but the coupling of the dilaton field to Standard Model particles is not universal, usually depending on their quark content [53–55].

## Code Availability Statement

This manuscript has no associated code/software. [Author’s comment: The methods and protocols are fully described in the manuscript and supporting documents.]
